# Correlation between locally versus centrally processed serum procalcitonin during emergency department research evaluation of febrile infants aged 0–60 days^☆^

**DOI:** 10.1016/j.plabm.2024.e00391

**Published:** 2024-03-22

**Authors:** Cosby G. Arnold, Prashant Mahajan, Russell K. Banks, John M. VanBuren, Nam K. Tran, Octavio Ramilo, Nathan Kuppermann

**Affiliations:** a Department of Emergency Medicine, University of California, Davis, School of Medicine, 2315 Stockton Blvd, PSSB Ste 2100, Sacramento, CA, 95817, USA; b Departments of Emergency Medicine and Pediatrics, University of Michigan Medical School, 1540 East Hospital Drive, CW 2-737, SPC 4260, Ann Arbor, MI, 48109-4260, USA; c University of Utah Data Coordinating Center, 295 Chipeta Way, Salt Lake City, UT, 84108, USA; d Department of Pathology and Laboratory Medicine, University of California, Davis, School of Medicine, 4400 V Street, Sacramento, CA, 95817, USA; e Department of Infectious Diseases, MS 320, Room E8050, St. Jude Children's Research Hospital, 262 Danny Thomas Place, Memphis, TN, 38105, USA; f Departments of Emergency Medicine and Pediatrics, University of California, Davis, School of Medicine, 2315 Stockton Blvd, PSSB Ste 2100, Sacramento, CA, 95817, USA

**Keywords:** Procalcitonin, Biomarkers, Serious bacterial infection, Infectious disease, Interlaboratory performance, Febrile infant

## Abstract

**Introduction:**

Procalcitonin (PCT) is a useful biomarker in the initial evaluation of febrile infants for serious bacterial infections (SBIs). However, PCT is not always available locally and must at times be frozen and shipped to a reference laboratory for research studies. We sought to compare PCT measured locally versus centrally at a reference laboratory during a research study.

**Materials and methods:**

This was a secondary analysis of a multicenter study of febrile infants ≤60 days evaluated for SBIs from June 2016 to April 2019. A PCT cutoff value of 0.5 ng/mL was used to stratify infants at low-versus high-risk of SBIs. Statistical analyses consisted of Spearman's correlation, Bland-Altman difference plotting, Passing-Bablok regression, Deming regression, and Fisher's exact testing at the 0.5 ng/mL threshold.

**Results:**

241 febrile infants had PCT levels measured both locally and at the reference laboratory. PCT levels measured locally on 5 different platforms and from the frozen research samples demonstrated strong Spearman's correlation (ρ = 0.83) and had similar mean PCT values with an average relative difference of 0.02%. Eleven infants with SBIs had PCT values < 0.5 ng/mL in both the clinical and research samples. Six other infants had differences in SBI prediction based on PCT values at the 0.5 ng/mL threshold between the clinical and research platforms.

**Conclusions:**

We found no significant differences in detection of febrile infants at high risk for SBI based on locally (on multiple platforms) versus centrally processed PCT. Testing at a central reference laboratory after freezing and shipping is an accurate and reliable alternative for research studies or when rapid turnaround is not required.

## Highlights

• Procalcitonin (PCT) is an important biomarker for serious bacterial infection (SBI).
• Multiple PCT assays strongly correlated with a single reference laboratory assay.
• Strong performance remained at the 0.5 ng/mL clinical decision threshold for SBI.
• Use of a central laboratory is reasonable when analyses cannot be performed locally.

## 1 Introduction

Serum procalcitonin (PCT) is the most accurate biomarker available for clinical prediction rules for emergency department (ED) identification of serious bacterial infections (SBIs; urinary tract infections [UTIs], bacterial meningitis, and bacteremia) in febrile infants ≤60 days old [1,2]. PCT outperforms C-reactive protein (CRP), absolute neutrophil count (ANC), and white blood cell count (WBC) in the identification of febrile infants with bacteremia and bacterial meningitis [3]. However, not all EDs have PCT available [4]. As a result, some EDs must freeze and ship blood samples for testing at central, reference laboratories.

We studied the performance of PCT measured locally with fresh samples on multiple platforms versus frozen samples shipped to a central laboratory for a research study predicting risk for SBI based on a defined measurement PCT threshold of 0.5 ng/mL. This threshold was derived from a research study for the development of the Pediatric Emergency Care Applied Research Network (PECARN) Febrile Infant Prediction Rule on infants ≤60 days old [2]. For the current study, blood samples were obtained from a prospectively enrolled cohort of febrile infants ≤60 days old [5]. Research PCT samples were tested at a central laboratory after freezing and shipping, and some hospitals also tested for PCT locally in their clinical laboratories as part of routine care. Agreement at a defined threshold for SBI risk between PCT measured locally versus centrally could provide insights regarding the reliability of results.

## 2 Methods

Febrile infants ≤60 days old were enrolled in a prospective observational multicenter (18 EDs) study in PECARN designed to evaluate the feasibility of RNA microarray analysis for diagnosis of SBI, defined as UTI, bacteremia, or bacterial meningitis. UTI was defined as growth of a known urinary pathogen with colony-forming units [CFU]/mL meeting one of three criteria: (1) ≥ 1000 CFU/mL for urine cultures obtained via suprapubic aspiration, (2) ≥ 50,000 CFU/mL via catheterization, (3) ≥ 10,000 CFU/mL via catheterized specimen in association with an abnormal urinalysis. We defined abnormal urinalysis as a urine dipstick test positive for leukocyte esterase or nitrite, or >5 WBCs per high power field. Bacteremia and bacterial meningitis were defined by growth of a known pathogen [6]. The methods for the parent study are detailed elsewhere [6]. Twelve out of the 18 participating sites reported clinical PCT in their EDs during the study period. The study was approved by the institutional review board at each site. Only a portion of infants from the parent study enrolled from June 2016 to April 2019 had results from both local and centrally processed PCT samples available. Blood for processing PCT for research measurements was centrifuged and stored at −80C within 6 h of blood draw, shipped to a central laboratory, and tested in batches monthly. All PCT tests at the central laboratory were performed with the Roche Diagnostics Elecsys® BRAHMS procalcitonin assay on the Cobas e411 immunoanalyzer. In a subset of these patients, PCT levels were also measured locally, and these levels were used as comparators with the centrally processed samples. Blood for locally processed PCT was collected and processed in adherence to institutional laboratory requirements. All sites were Clinical Laboratory Improvement Amendment (CLIA) certified. Platforms used for local measurement across sites were: Elecsys BRAHMS PCT on Cobas E601/602, VITROS BRAHMS PCT on VITROS 5600, VIDAS BRAHMS PCT on VIDAS, Architect BRAHMS PCT on Architect i2000, and ATELLICA BRAHMS PCT on ATELLICA IM 1300 (please see Supplemental Table 1 for details). A PCT cutoff value of 0.5 ng/mL was used to stratify low-versus high-risk thresholds for SBI. Spearman's rank correlation coefficient, Bland-Altman difference plot, Passing-Bablok regression, Deming regression and Fisher's exact test were used for statistical analyses.

## 3 Results

**Fig. 1:**
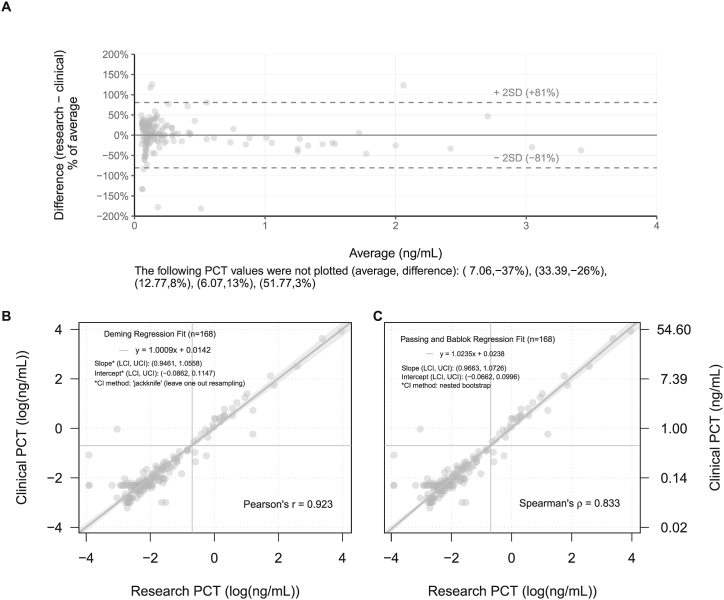
Results obtained with different procalcitonin (PCT) assays compared to a central reference laboratory: (A) Bland-Altman difference plot; (B) Deming regression; and (C) Passing-Bablok regression.

A total of 241 febrile infants from 12 sites had PCT levels measured both at the local site for clinical care and frozen and shipped to a central laboratory for research analyses. A total of 21 infants (8%) had documented SBIs; 17 infants (7%) had UTIs alone, 3 (1%) had bacteremia alone, and 1 had both bacteremia and bacterial meningitis. For regression analysis and Bland-Altman difference plotting, we excluded the 73 febrile infants from two sites whose lowest threshold for reporting PCT was <0.5 ng/mL rather than exact values below this threshold. In the remaining 168 infants, Bland-Altman plots demonstrated an average relative difference of 0.02% (±2SD, −81% to +81%) between the clinical and research samples (Fig. 1A). We also found a strong correlation between the PCT levels measured from the frozen research samples and locally performed PCT levels (Spearman's ρ = 0.83; Pearson's r = 0.92; Fig. 1B and C). Because the confidence intervals resulting from both Deming and Passing-Bablok fitted regressions contain 1 and 0 respectively, we lack statistical evidence to suggest proportional or systematic differences between research and clinical PCT testing methods. Spearman's correlation should be considered superior to Pearson's correlation due to the skewness of the data. Given prior reports of bias between platforms [7], we performed a platform-adjusted analysis and found the strength and direction of the correlation to be unaffected.

Overall agreement between clinical and research samples at the 0.5 ng/mL threshold was very high (p < 0.001). Of the 241 infants, 207 were classified as low risk by both methods and 28 were classified as high risk by both methods. Eleven infants with SBIs were missed at the 0.5 ng/mL threshold by both the clinical sample and the research sample (8 with UTIs; 3 with bacteremia caused by *E. coli*, Group B strep, and *S. aureus*). Only 6 (2.5%) of the 241 infants had differences in SBI prediction between the local and research samples based on PCT values at the 0.5 ng/mL threshold. Four of the 6 infants did not have SBIs; 3 of the 4 were identified as high-risk by the research sample and 1 was identified as high-risk by the clinical sample. Of the 2 discrepantly classified infants with SBIs, both were identified as high-risk by the research sample but not by the clinical sample (Table 1). One had an *E.coli* UTI and the other had *S. pyogenes* bacteremia.

**Table 1:** Clinical characteristics of infants with discordant PCT levels between locally and centrally processed blood samples at the 0.5 ng/mL threshold.

Clinical site #	Age	Qualifying temperature (Celsius)	Yale observation scale	Absolute neutrophil count (1000 per microliter)	Clinical assessment of SBI risk	UTI	Bacteremia	Meningitis	Clinical site procalcitonin	Clinical site procalcitonin measurement assay	Research procalcitonin
7	≤28 days	39.1	6	7.2	6–10%	No	No	No	<0.5	Biomerieux VIDAS BRAHMS PCT	0.504
6	>28 days	39.5	6	4.1	6–10%	No	No	No	<0.5	Abbott Architect BRAHMS PCT	0.586
8	>28 days	39.5	6	11.4	1–5%	No	No	No	0.38	Biomerieux VIDAS BRAHMS PCT	0.543
1	>28 days	38.4	6	3.5	1–5%	No	No	No	0.97	Roche Elecsys BRAHMS PCT	0.048
10	>28 days	38.6	8	4.4	1–5%	Yes (*E. coli*^a^*)*	No	No	0.33	Roche Elecsys BRAHMS PCT	0.778
10	>28 days	38.6	6	5.2	<1%	Unable to classify	Yes *(S. pyogenes*^b^*)*	No	0.26	Roche Elecsys BRAHMS PCT	0.553

a Escherichia coli.

b Streptococcus pyogenes.

## 4 Discussion

We found a strong correlation in PCT levels in blood samples from young febrile infants processed locally at the clinical site from multiple platforms and those processed, frozen and shipped to a central research laboratory. Although PCT is a highly accurate biomarker, it is not perfect when used in isolation. Both the clinical and research PCT levels were normal in 11 febrile infants with SBIs. Additionally, differences in PCT levels at the 0.5 ng/mL threshold occurred in two infants with SBIs, both of whom were identified as high-risk by the research sample but not the clinical sample.

To our knowledge, our study is the first to evaluate PCT performance across multiple measurement platforms in pediatric emergency care [8] and demonstrates feasibility for clinical research studies conducted at multiple sites with different testing platforms given the high correlation with centrally processed samples. Our findings demonstrate PCT values obtained using multiple assays in multiple EDs were highly correlated and had good performance at the prespecified clinical decision threshold of 0.5 ng/mL across many immunoassays, and had good correlation with the frozen, shipped samples processed centrally. Our findings also suggest, based on PCT results from multiple centers, that blood samples frozen and shipped and measured on a central analyzer can be used for risk stratification when integrated with other routinely available clinical data [2].

Because PCT results are not routinely available at all EDs across the United States, blood must frequently be frozen and shipped for research purposes [4]. This is of clinical importance, when PCT levels are not needed immediately, because PCT is a precise biomarker in several conditions including older children with pneumonia [9,10], and plays an important role in antimicrobial stewardship [11]. In this study, we investigated the performance of PCT at the 0.5 ng/mL threshold as this threshold is used to risk-stratify febrile infants for SBIs [1,2,4].

Our study has limitations. Because two EDs reported the lower cutoff of 0.5 ng/mL rather than the actual raw low values, we could not evaluate the correlation of exact PCT levels between all research and clinical samples. Nevertheless, PCT levels in the clinical samples were highly associated both when measured as an exact value and when considered at the 0.5 ng/mL threshold (including all samples). Although only 241 infants out of the total initial cohort were included in our investigation, the rates of SBIs were similar to those reported for young febrile infants in previous studies [2,[12], [13], [14]].

In conclusion, clinical samples for PCT processed on multiple platforms and frozen, shipped research samples processed on one platform had similar PCT levels. However, turnaround time of shipped frozen samples is not sufficiently rapid to affect clinical decision making in the emergency department. Therefore, these findings suggest that freezing and shipping blood samples to a central reference laboratory is an accurate and reliable alternative for research studies or when rapid turnaround is not critical and analyses cannot be performed locally.

## Funding/support

This study was supported by grant R01HD085233 from the 10.13039/100009633Eunice Kennedy Shriver National Institute of Child Health and Human Development of the 10.13039/100000002National Institutes of Health. This project was also supported in part by the 10.13039/100000102Health Resources and Services Administration (HRSA) of the 10.13039/100000016U.S. Department of Health and Human Services (HHS), the Emergency Medical Services for Children (EMSC) program through the following grants: DCC-10.13039/100007747University of Utah (U03MC00008), GLACiER-Nationwide Children's Hospital (U03MC00003), HOMERUN-Cincinnati Children's Hospital Medical Center (U03 MC22684), PEMNEWS-Columbia University Medical Center (U03MC00007), PRIME-University of California at Davis Medical Center (U03MC00001), SW NODE-University of Arizona Health Sciences Center (U03 MC28845), WBCARN-Children's National Medical Center (U03MC00006). This information or content and conclusions are those of the authors and should not be construed as the official position or policy of, nor should any endorsements be inferred by HRSA, HHS or the U.S. Government.

## Additional contributions

The authors thank the PECARN Steering Committee members, the research coordinators in PECARN and the project staff at the EMSC Data Center at the University of Utah. No compensation was received from a funding sponsor for these contributions.

## Other participating sites and site principal investigators

1. Aaron Leetch, MD, Department of Emergency Medicine and Pediatrics, College of Medicine Tucson, University of Arizona, ALeetch@aemrc.arizona.edu
2. Lise E. Nigrovic, MD, MPH Department of Pediatrics, Boston Children's Hospital, Harvard Medical School, lise.nigrovic@childrens.harvard.edu
3. Peter S. Dayan, MD, MSc, Division of Pediatric Emergency Medicine, Department of Emergency Medicine, Columbia University Vagelos College of Physicians & Surgeons, psd6@cumc.columbia.edu
4. Fran Balamuth, MD, Division of Emergency Medicine, Department of Pediatrics, Children's Hospital of Philadelphia, balamuthf@email.chop.edu
5. Angela Ellison, MD, Division of Emergency Medicine, Department of Pediatrics, Children's Hospital of Philadelphia, ellisona@email.chop.edu
6. Richard M. Ruddy, MD, Division of Emergency Medicine, Cincinnati Children's Hospital Medical Center, Department of Pediatrics, University of Cincinnati College of Medicine, richard.ruddy@cchmc.org
7. Shireen M. Atabaki, MD, MPH, Division of Emergency Medicine, Department of Pediatrics, Children's National Medical Center, The George Washington School of Medicine and Health Sciences, satabaki@cnmc.org
8. Rakesh Mistry, MD, MS, Department of Pediatrics, Yale University School of Medicine, Yale-New Haven Children's Hospital, Rakesh.Mistry@yale.edu *Formerly: Department of Pediatrics, University of Colorado School of Medicine, Colorado Children's Hospital*
9. Elizabeth C. Powell, MD, MPH, Division of Emergency Medicine, Department of Pediatrics, Ann & Robert H. Lurie Children's Hospital, Northwestern University Feinberg School of Medicine, epowell@luriechildrens.org
10. Michelle L. Pickett, MD, MS, Division of Pediatric Emergency Medicine, Department of Pediatrics, Medical College of Wisconsin, mpickett@mcw.edu
11. Grace Park, DO, MPH, Department of Emergency Medicine, Pediatric Emergency Medicine, The University of New Mexico, GPark@salud.unm.edu
12. Daniel M. Cohen, MD, Section of Emergency Medicine, Department of Pediatrics, Nationwide Children's Hospital and The Ohio State University, daniel.cohen@nationwidechildrens.org
13. Amanda Bogie, MD, FAAP, Section of Emergency Medicine, Department of Pediatrics, The University of Oklahoma College of Medicine, Amanda-Bogie@ouhsc.edu
14. Eric W. Glissmeyer, MD, MBA, Department of Pediatrics, Primary Children's Hospital, University of Utah, eric.glissmeyer@hsc.utah.edu
15. Andrea T. Cruz, MD, MPH, Divisions of Emergency Medicine and Infectious Diseases, Department of Pediatrics, Texas Children's Hospital, Baylor College of Medicine, acruz@bcm.edu
16. Leah Tzimenatos, MD, Department of Emergency Medicine, University of California, Davis School of Medicine, lstzimenatos@ucdavis.edu
17. Allison Cator, MD, PhD, Departments of Emergency Medicine and Pediatrics, University of Michigan, acator@med.umich.edu
18. Robert Hickey, MD, Division of Pediatric Emergency Medicine, Department of Pediatrics, Children's Hospital of Pittsburgh of UPMC, University of Pittsburgh School of Medicine, Robert.Hickey@chp.edu
19. Melissa Vitale, MD, Division of Pediatric Emergency Medicine, Department of Pediatrics, Children's Hospital of Pittsburgh of UPMC, University of Pittsburgh School of Medicine, melissa.vitale@chp.edu
20. Kimberly S. Quayle, MD, Department of Pediatrics, St. Louis Children's Hospital, Washington University School of Medicine, quayleks@wustl.edu

## CRediT authorship contribution statement

**Cosby G. Arnold:** Writing – review & editing, Writing – original draft. **Prashant Mahajan:** Writing – review & editing, Data curation. **Russell K. Banks:** Methodology, Formal analysis. **John M. VanBuren:** Methodology, Formal analysis. **Nam K. Tran:** Writing – review & editing, Methodology. **Octavio Ramilo:** Writing – review & editing, Data curation. **Nathan Kuppermann:** Writing – review & editing, Writing – original draft, Methodology, Data curation, Conceptualization.

## Declaration of competing interest

The following authors have the following conflicts to disclose.

N.K. is funded by the NIH and HRSA.

O.R. has received research grants to institution from Janssen, Merck, NIH and the Bill & Melinda Gates foundation; and fees for participation in Advisory Boards from Sanofi-Pasteur, Merck, and Pfizer and for lectures from Pfizer, Sanofi-Pasteur and Astra-Zeneca.

N.T. has received fees for consulting for respiratory molecular testing and honoraria for multiplex respiratory pathogen testing and data informatics from Roche Diagnostics.

C.G.A., P.M., and J.M.V. have no potential conflicts of interest to disclose.

## Data Availability

Data will be made available on request.
