# Supplementary material for: Correlation between locally versus centrally processed serum procalcitonin during emergency department research evaluation of febrile infants aged 0–60 days
Source: Pract Lab Med. 2024 Mar 22;39:e00391. doi: 10.1016/j.plabm.2024.e00391 (PMC11075055; doi:10.1016/j.plabm.2024.e00391)
Supplement: Multimedia component 1 [file mmc1.docx]

| **Site #** | **Instrument** | **Company** | **Method** | **Assay Principle** | **Analytical Measurement Range (ng/mL)** |
| --- | --- | --- | --- | --- | --- |
| 1 | Cobas E601/602 | Roche Diagnostics, Indianapolis, IN, USA | Elecsys BRAHMS PCT | Electrochemiluminescence immnoassay (ECLIA) | 0.02-100 |
| 2 | VITROS 5600 | Ortho Clinical Diagnostics, Raritan, New Jersey, USA | VITROS BRAHMS PCT | Chemiluminescence immunoassay (CLIA) | 0.03-1000 |
| 3 | VIDAS  6/20/19 - | Biomerieux, Marcy L'Etoile, France | VIDAS BRAHMS PCT | Enzyme-linked fluorescent immunoassay (ELFA) | 0.05-200 |
|  | Cobas  Unknown if E601/602 or E411  2018 – 6/20/19 | Roche Diagnostics, Indianapolis, IN, USA | Elecsys BRAHMS PCT | Electrochemiluminescence immnoassay (ECLIA) | 0.02-100 |
|  | Unknown  Before 2018 | Unknown | Unknown | Unknown | Unknown |
| 4 | VIDAS | Biomerieux, Marcy L'Etoile, France | VIDAS BRAHMS PCT | Enzyme-linked fluorescent immunoassay (ELFA) | 0.05-200 |
| 5 | Architect i2000 | Abbott Laboratories, Chicago, IL, USA | Architect BRAHMS PCT | Chemiluminescent microparticle immunoassay (CMIA) | 0.02-100 |
| 6 | VIDAS | Biomerieux, Marcy L'Etoile, France | VIDAS BRAHMS PCT | Enzyme-linked fluorescent immunoassay (ELFA) | 0.05-200 |
|  | Architect i2000 | Abbott Laboratories, Chicago, IL, USA | Architect BRAHMS PCT | Chemiluminescent microparticle immunoassay (CMIA) | 0.02-100 |
| 7 | VIDAS | Biomerieux, Marcy L'Etoile, France | VIDAS BRAHMS PCT | Enzyme-linked fluorescent immunoassay (ELFA) | 0.05-200 |
| 8 | VIDAS | Biomerieux, Marcy L'Etoile, France | VIDAS BRAHMS PCT | Enzyme-linked fluorescent immunoassay (ELFA) | 0.05-200 |
|  | ATELLICA IM 1300 | Siemens Healthcare Diagnostics, Berkeley, CA, USA | ATELLICA IM BRAHMS PCT | Chemiluminescent microparticle immunoassay (CMIA) | 0.04-50 |
| 9 | VIDAS | Biomerieux, Marcy L'Etoile, France | VIDAS BRAHMS PCT | Enzyme-linked fluorescent immunoassay (ELFA) | 0.05-200 |
| 10 | Cobas E601/602 | Roche Diagnostics, Indianapolis, IN, USA | Elecsys BRAHMS PCT | Electrochemiluminescence immnoassay (ECLIA) | 0.02-100 |
| 11 | VIDAS | Biomerieux, Marcy L'Etoile, France | VIDAS BRAHMS PCT | Enzyme-linked fluorescent immunoassay (ELFA) | 0.05-200 |
| 12 | Cobas E411 | Roche Diagnostics, Indianapolis, IN, USA | Elecsys BRAHMS PCT | Electrochemiluminescence immnoassay (ECLIA) | 0.02-100 |

**Supplemental Table 1.** Description of instruments, methods, and assay principles used in this multi-center study.
